# Demographic and spatial study of visceral leishmaniasis in the state of Alagoas, Brazil, during 2007-2018

**DOI:** 10.1590/0037-8682-0610-2020

**Published:** 2021-02-10

**Authors:** Beatriz Maria de Almeida Braz, Raizza Barros Sousa Silva, Suzanna Cavalcante Lins, Diego Ricardo Xavier Silva, Walter Massa Ramalho, Marcia Almeida de Melo

**Affiliations:** 1 Universidade Federal de Campina Grande, Laboratório de Biologia Molecular do Semiárido, Programa de Pós-Graduação em Ciência e Saúde Animal (PPGCSA), Patos, PB, Brasil.; 2 Fundação Oswaldo Cruz, Instituto de Comunicação e Informação Científica Tecnológica, Rio de Janeiro, RJ, Brasil.; 3 Universidade de Brasília, Faculdade de Ceilândia, Brasília, DF, Brasil.

**Keywords:** Epidemiology, Georeferencing, Incidence, Epidemiological surveillance

## Abstract

**INTRODUCTION:**

Visceral leishmaniasis has a broad worldwide distribution and constitutes a public health problem in the Northeast of Brazil. Located in this region is the state of Alagoas, where the disease is endemic in humans and where there has been a significant increase in the number of positive dogs. The objective of this study was to describe the temporal and spatial distribution of the cases of human VL in the state of Alagoas with the aim of identifying transmission risk areas in the period from 2007 to 2018.

**METHODS:**

The data available in the National Disease Notification System (SINAN-NET) were used. The Bayesian incidence rate and the Moran’s global index were calculated using the Terra View 4.2*.*2 program, and the maps were created using QGIS*2.18.0*.

**RESULTS:**

From the 102 municipalities, 68.6% (n= 70) had at least one notified case of VL in the years of study. A total of 489 cases were registered, with an average of 40.7 cases per year and an incidence rate of 1.25/100,000 inhabitants. The highest number of confirmed cases (105) occurred in 2018. Male individuals and children between 1-4 years old were the most affected, and 64% of the cases were in rural areas. Spatial dependence was detected in all the intervals except for the first triennium, and clusters were formed in the west of the state.

**CONCLUSIONS:**

Alagoas presented an accentuated geographical expansion of VL, and it is necessary to prioritize areas and increase surveillance actions and epidemiological control.

## INTRODUCTION

Visceral leishmaniasis (VL) is a neglected tropical disease caused by different species of the genus *Leishmania* ^1^ and transmitted through the blood repast of females of the *Lutzomyia (Lutzomyia) longipalpis* ^2^ species. In Brazil, the specie *Leishmania infantum* is the etiological agent. The species *Lutzomyia longipalpis* and *Lutzomyia cruzi* are the vectors related to transmission^3^ ^,^ ^4^. Dogs (*Canis lupus familiaris*) are the main domestic reservoirs and play a major role in the maintenance of the transmission cycle between humans and the vectors^5^. 

Human visceral leishmaniasis (HVL) has a broad worldwide distribution with occurrence in Asia, Europe, Middle East, Africa, and in at least 12 countries of the Americas^4^, where from the reported cases came from deforested, peripheral areas or those in the process of territorial expansion^6^. The disease is endemic in Brazil, representing more than half of the cases of the Americas, and is distributed in the North, Central-West, Northeast, and Southeast regions, representing a public health problem^7^ ^,^ ^8^.

Situated in the Northeast region, the state of Alagoas had the first cases of VL notified in 1934, originating from the coastal region and *Zona da Mata* (forest region)^9^. The disease is endemic in the intermediate geographical regions of Maceió and Arapiraca, an area composed of 66 municipalities^10^, where there has been a large increase in the number of confirmed cases over the last ten years^11^ ^,^ ^12^. In addition, there was a significant increase in the number of dogs who were diagnosed as positive for the using serological methods. This resulted in the euthanasia of 909 positive animals in 2018^13^.

To date, besides the prevention measures aimed at the population at risk and the vector, the control strategy implemented by the Brazilian Health Ministry, through its Visceral Leishmaniasis Surveillance and Control Program (VLSCP), is the euthanasia of dogs with two positive serological or parasitological results^14^. 

Studies, which analyze the expansion process of the VL and the spatial and temporal variation of the incidence, are of great importance due to its dispersion in Brazil over the recent years^14^. Therefore, it is necessary to process and use the information available in public databases, including the National Disease Notification System (SINAN-NET) and the Geographic Information System (GIS), as health surveillance tools for the reassessment of endemic control programs. Thus, the objective of this study was to describe the temporal and spatial distribution of cases of VL in the state of Alagoas, to identify the areas with high risk of transmission of the disease in the period from 2007 to 2018, in triennial intervals. 

## METHODS

Alagoas is one of the 27 federative units of Brazil. Located in the Northeast region, it has 102 municipalities and a population of approximately 3,322,000 inhabitants in a territorial area of 27,778,506 km². Until 2017, the state was divided into three mesoregions according to the climate, each composed of municipalities located in the Agreste, Sertão, and Eastern regions of the state of Alagoas; the latter covers the municipalities of the coast and the forest zone. The Brazilian Institute of Geography and Statistics (IBGE) subsequently substituted this division with two intermediate geographical regions Maceió and Arapiraca, and 11 immediate regions^15^.

In this study, a descriptive evaluation with a temporal and spatial analysis of the new notified and confirmed cases was conducted using the National Disease Notification System (SINAN-NET) on the website of the National Health System Information Technology Department (DATASUS) between 2007 and 2018; categorized in four triennial intervals: 2007-2009, 2010-2012, 2013-2015, and 2016-2018. The data collection was carried out in February 2020. The parameters collected were age, sex, level of education, zone of residence, and evolution of the disease and these were presented as relative and absolute frequencies.

The cartographic network of the state was obtained from the website of the IBGE and the data was inserted into Microsoft Excel 2013® spreadsheets in which the cumulative gross rate of incidence/municipality was calculated and imported to the Terra View 4.2.2 software. To calculate the smoothed incidence rate of each municipality, a local empirical Bayesian estimator was used. This uses the incidence rates of the neighboring municipalities, converging them to a local average, with the objective of correcting the generated rates, making them less unstable. The intervals of the incidence rates considered were the quartile, average, median, minimum and maximum values.

The different classes of incidence rates were categorized by adopting a quartile-based legend. To understand the spatial association patterns (cluster*s*) and verify the extreme values (outliers), the Moran’s global index was used. This informed the level of spatial interdependence, varying from -1 to 1^16^. The descriptive significance levels of the clusters were obtained from Moran’s Global Index, with a 0.05 level of significance. Being adopted. *P-*values ≤0.05, indicated spatial dependence and was demonstrated by Moran’s Map. The municipalities that were a high priority for interventions, were then identified. The QGIS 2.18.0 software was used for the elaboration of the thematic maps.

## RESULTS

Between 2007 and 2018 489 cases of HVL were registered in the state of Alagoas, with an average of 40.7 cases per year and an incidence of 1.25/100 per thousand inhabitants. Of the 102 municipalities of the state, 68.6% (n= 70) notified at least one case of HVL in the 12 years of study. In the first triennium (2007-2009), ninety-one (91) cases were notified in 37.2% (n= 38) of the municipalities, corresponding to 0.98 cases/100 thousand inhabitants. In the second (2010-2012) and third trienniums (2013-2015) there was a small increase to 106 (1.12 cases/100 thousand inhabitants) and 115 cases (1.15 cases/100 thousand inhabitants), in 39.2% (n= 40) and 40.1% (n= 41) of the municipalities, respectively. In the fourth triennium (2016-2018), there was a significant increase to 177 cases (1.76 cases/100 thousand inhabitants), distributed in 41.1% (n=42) of the cities. 

Most of the individuals affected were male (66.2%) and children aged between 1 and 4 years (28.6%), 87.7% of the cases were autochthonous and 64% of the total cases originated from rural areas. The fatality rate of VL was 7.7% (38), with an average of 3.16 cases/100 thousand inhabitants, while the recovery rate was 46.21% (Table 1). 

**TABLE 1:** Epidemiological variables of the cases of HVL in the state of Alagoas, from 2007 to 2018.

Variable	Number	Percentage
**Sex**		
Male	324	66.2
Female	165	33.7
**Age**		
<1 year	25	5.1
1-4 years	140	28.6
5-9 years	66	13.4
10-14 years	48	9.8
15-39	40	8.1
20-39	112	22.9
40-59	46	9.4
60-79	12	2.4
**Origin of the cases**		
Autochthonous	429	87.7
**Variable**	**Number**	**Percentage**
Non-autochthonous	20	4
Indeterminate	40	8.1
**Zone of residence**		
Urban	132	26.9
Rural	313	64
Peri-urban	35	7.1
Ignored/blank	9	1.8
**Evolution**		
Ignored/blank	162	33.1
Cure	226	46.2
Abandonment	3	0.6
Death due to VL	38	7.7
Death due to another cause	14	2.8
Transference	46	9.4

**FIGURE 1:**
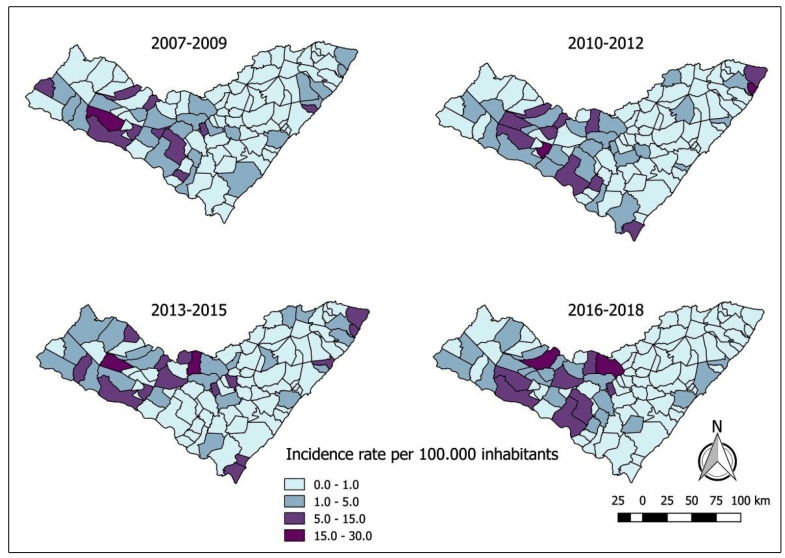
Incidence rate of human visceral leishmaniasis per municipality of Alagoas, Brazil, from 2007 to 2018 in triennial intervals. Data source: National Disease Notification System - SINAN.

**FIGURE 2:**
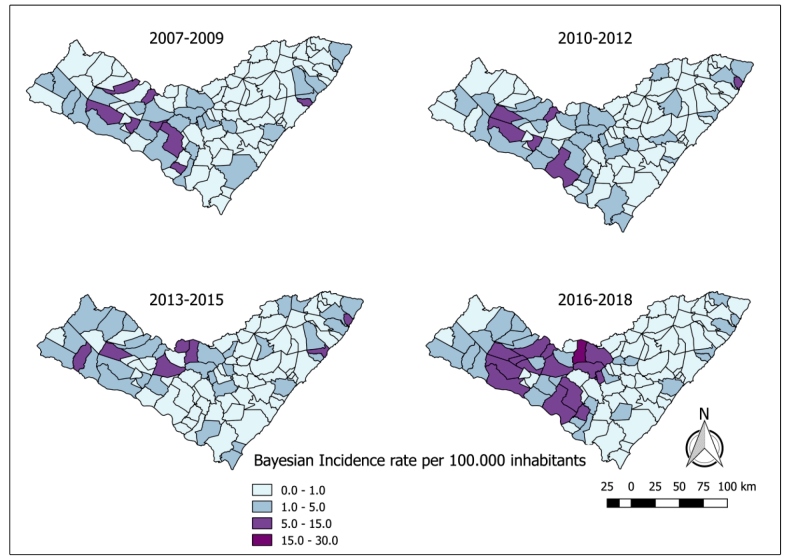
Bayesian incidence rate of human visceral leishmaniasis per municipality of Alagoas, Brazil, from 2007 to 2018 in triennial intervals. Data source: National Disease Notification System - SINAN.

The incidence map showed that there were cases of the disease in all the geographical areas of the state during the four triennial intervals; however, the highest incidence rates were concentrated in the first triennium (15 to 30/100.000 inhabitants), in municipalities of the center-west, with isolated cases in the east of the state (Figure 1). This was confirmed using a Bayesian map of smoothed cumulative incidence in the same period of the study (Figures 2).

Although a similar distribution occurred in the second and third trienniums, high incidence rates were found in municipalities on the coast of the state (15 to 30/100,000 inhabitants), there was a decline in the fourth interval of the study (1 to 5/100,000 inhabitants). The cases in the Center-West region; however, showed an increase of this rate when compared to the previous years (15 to 30/100.000 inhabitants).

**FIGURE 3:**
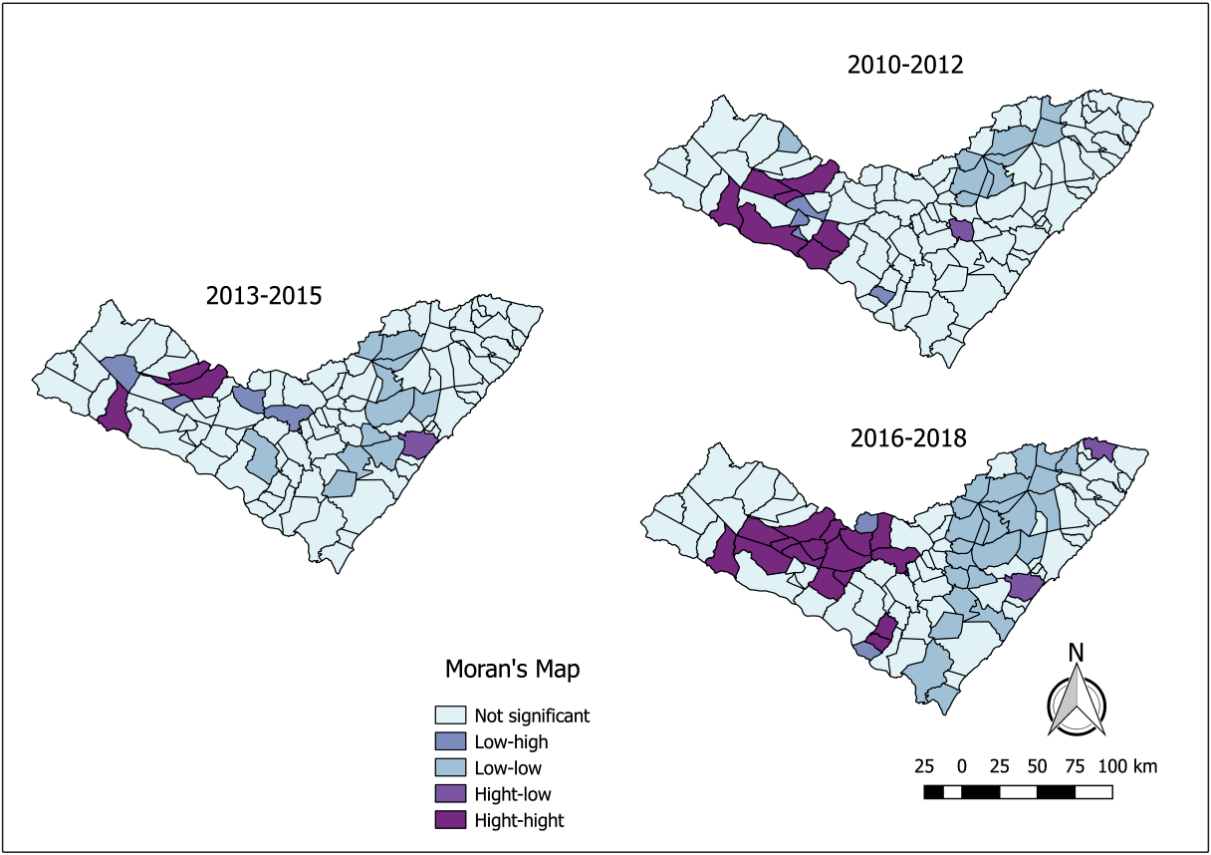
Moran’s map for human visceral leishmaniasis per municipality of Alagoas, Brazil, from 2007 to 2018 in triennial intervals. Data source: National Disease Notification System - SINAN.

The Moran’s index showed that there was no spatial dependence in the first triennium and, therefore there were no significant clusters; however, a spatial dependence was observed in the second (*P*= 0.015), third (*P*= 0,009) and fourth trienniums (*P*= 0.002) with a similarity between the municipalities, although the correlation was weak (Table 2). Based on this index, a Moran’s Map was drawn (Figure 3). In the second triennium, a high incidence cluster was identified in cities near the municipalities of Monteirópolis and Olho D’Água das Flores, but these presented a low-risk cluster. 

**TABLE 2:** Moran´s index values and the *P*-values for each triennium studied in the state of Alagoas.

Trienniums	Moran’s index	p - value*
2007 - 2009	0.076	0.12
2010 - 2012	0.182	0.015*
2013 - 2015	0.190	0.009*
2016- 2018	0.336	0.002*

*P-value ≤0.05 indicates spatial dependence.

In the East region, in the municipalities of the *zona da mata* (forest region), an agglomerate was formed in the low-low category. During this period, even though it was between the municipalities with low risk of occurrence of the disease, the Anadia municipality stood out as it presented a significant cluster.

In the third triennium, there was a reduction in the number of significant clusters, which were situated in the semi-arid municipalities and even though it was close to the risk region, the municipality of Carneiros presented a low-risk cluster. In the same study interval, a cluster of the high-low category was observed in the metropolitan region of Maceió, the capital of the state. In the fourth triennium, a highly significant cluster was formed west of the state of Alagoas, within the limits of the municipality of Minador do Negrão, as well as another, close to São Braz, but, despite this, they did not present a high risk for the occurrence of the disease. In this triennium, a low-incidence cluster was also observed, starting from the central-south to the east, surrounding the municipalities of Jacuípe to the northeast and in Marechal Deodoro, to the southeast of the state.

## DISCUSSION

The present study, the first study of this nature in the state, reported the temporal and spatial distribution of new cases of VL in the state of Alagoas with the data being analyzed in triennial intervals, from 2007 to 2018. The incidence data revealed a progressive geographical expansion of HVL in the period under study, showing a tendency to persist in municipalities which already had notified cases, strengthening the endemic status of the Alagoas. 

According to Silveira et al.^17^and Rocha et al.^18^, the disease is predominantly rural, and has been constantly registered in municipalities of the *Agreste* and *Sertão* regions, such as Arapiraca, Palmeira dos Índios, Traipú, Cacimbinhas, Igaci, Santana do Ipanema and São José da Tapera. In this study, Palmeira dos Índios and São José da Tapera, situated in the immediate geographical region of Arapiraca, presented the highest number of cases of the disease, and over the years has presented a status of moderate and intense transmission, respectively^18^. These municipalities have average (0.638) and low (0.527) Municipal Human Development Indexes (IDH-M)^14^ and the cases tended to progressively increase in each triennium in Palmeira dos Índios and to decrease in São José da Tapera, which may be an indication of the lack of an early diagnosis, shortcomings in the epidemiological surveillance and sub-notification levels.

The occurrence of VL may be also associated with the environmental conditions of each geographical region, and these must be taken into consideration. As described by Leite et al.^19^, in situations of high temperature and humidity, there was a decrease in the number of cases, in response to the low adaptation and reproduction of the vector. Additionally, Rocha et al.^18^justified that the emergence of cases in Alagoas may be due to socio-environmental issues, such as poor housing conditions, lack of basic sanitation and water supply, peridomicile waste accumulation both in urban and rural residences and, the presence of animal raising close to the houses and proximity to green areas. These factors interfere directly with the adaptation and reproduction of the vector^20^, as seen as in the *Lu. longipalpis* species, that was involved in the transmission of the VL in Brazil; it adapted completely to this region and can be found in all the territories of the state of Alagoas^21^ ^,^ ^22^ ^,^ ^23^. It is important to report that, where there was a record of cases, municipal health agents were trained to act in entomological surveillance and in other actions within the scope of VL control. However, most municipalities did not carry out these activities due to the lack of human resources^13^.

Most of the cases affected were children under the age of five and individuals of the male sex, a tendency also mentioned by other authors^17^ ^,^ ^24^ ^,^ ^25^ ^,^ ^26^. The children are more susceptible to the infection due to the immaturity of the cellular immune system^27^ ^,^ ^28^, that can be accentuated by malnutrition^29^. In Brazil, specifically in the Northeast, the *sertão* region is historically known for the uncertainties caused by droughts and poor housing and living conditions, where especially children live in conditions of malnutrition, favoring the prevalence and worsening of diseases^19^ ^,^ ^30^ ^,^ ^31^. In contrast, when children present with a good nutritional state and up-to-date immunizations, the incidence of VL is reduced^25^. With respect to individuals of the male sex, these have a greater tendency of developing occupational diseases^32^.

VL was reported up to the 1980s as a rural endemic disease in Brazil, but it known that the disease has gone through a process of expansion and started to present a status of urban and peri-urban transmission. This is due to several factors such as, the difficulties in identifying and eliminating the reservoirs, adaptation of the vector to the peridomicile, the high cost of the control actions, and insufficient control^21^ ^,^ ^29^. Despite this, in this study, more cases were observed to originate from rural areas, evidencing an old pattern of behavior of the disease in the state, arising from socio-economic and cultural issues of the populations of these areas, as well as low educational levels indicating a lack of knowledge of the disease, the presence of the canine reservoirs and uncertain sanitary conditions.

Of the individuals diagnosed, 46.21% recovered and 7.7% died from VL. In these cases, the precision in the diagnosis treatment may have reduced the fatalities; however, the operational difficulties in the basic healthcare network of the municipalities which prevent this^33^. However, it is also important to consider the percentage of ignored/blanks, equivalent to 33.12% of the total, may have compromised the exact the numbers of deaths and recoveries.

From the interpretation of the crude and smoothed incidence maps, it was evident that there was a heterogenous distribution and expansion into the interior of the state, with the emergence of new outbreaks in the east and the maintenance of the profile of active infections in former areas of occurrence in the *Sertão* region. The immediate geographical regions of Delmiro Gouveia, Santana do Ipanema and Pão de Açúcar - Olho D’Água das Flores - Batalha are situated in the driest area of the state territory, where the average annual rainfall is of 400 to 600 mm. The immediate geographical regions of Arapiraca and Palmeira dos Índios are situated between two distinct biomes, with small humid areas and wetlands, and precipitation varying between 600 mm and 900 mm^34^. 

According to Furtado et al.^35^ and Oliveira & Montoni^11^, these regions presented an elevation in the incidence over the years, as seen in the fourth triennium of this study However, these authors disagree with Pedrosa & Rocha^36^ when they state that the majority of the cases came from the immediate regions situated in the coastal stretch such as those of Maceió, Porto Calvo - São Luís do Quitunde and Penedo and, from the immediate regions of São Miguel dos Campos, União dos Palmares and Atalaia, restricted areas of Atlantic forest, where the climate is rainy tropical with a dry summer. In these non-endemic places, the presence of the disease was related to the migration of people and dogs from endemic areas^35^.

According to the Health Ministry^14^, the formation of clusters provided knowledge of the distribution of the disease and enabled the assessment of whether the occurrence was related to factors such as the presence of the vector and the migratory flow of individuals and infected humans and hence, the prioritization of the areas at risk. In this study, high risk clusters were identified in the Moran’s Map in the second, third and fourth trienniums, notably more prevalent in the municipalities of the *Sertão* region. However, it is also observed that, proximity to high risk clusters such as the municipalities of Monteirópolis, Olho D’água das Flores, Carneiros, Minador do Negrão, and São Braz presented a low risk for the occurrence of the disease.This may have been due to either surveillance and control of VL in these cities, not disregarding the possibility of increases in the incidence during the following years, or the presentation of particular bioclimatic characteristics that prevent the establishment of the cycle of the disease. In contrast, Anadia, Marechal Deodoro and Jacuípe, situated between the low-risk municipalities, presented risk clusters, which may be associated to the possible intrinsic factors of each municipality, such as those of the economic sphere, those associated to the migration of individuals, and to the non-execution of the measures of the Visceral Leishmaniasis Control Program. 

Considering the importance of the dog in the transmission chain of the VL^10^, it is important to mention that although Marechal Deodoro, a municipality situated on the coastal stretch and in the metropolitan region of Maceió, presented a low number of cases of the disease in humans, in 2018 confirmed 105 cases of canine visceral leishmaniasis (CVL), published by means of the Informative Notice n. 51/2018 of the Health Surveillance Superintendence (SUVISA)^13^. This points to the need for reviewing the measures of surveillance in regions, either without the occurrence or with few human cases, since there have been reports that the infections in dogs precede human infection; when a critical value of infected animals is reached^37^ ^,^ ^38^.

According to Anselin^39^, it is possible to visualize the spatial dependence between the municipalities using the Global Moran’s Index. This was verified in the second, third and fourth trienniums, but with a weak correlation between the municipalities, in other words, there were few similarities in the distribution of the cases. 

The Visceral Leishmaniasis Surveillance and Control Program (VLSCP)^14^ cites that measures restricted only to the municipalities which present cases of HVL and CVL have not proved to be effective in the control of the disease. Therefore, transmission or areas at risk must be better defined and municipalities and states without occurrence (or with silent occurrence) must also implement surveillance, with the aim of minimizing the consequences of zoonosis in areas without transmission.

Several factors can be associated to the spread of the disease in Alagoas, such as the disorderly occupation of the outskirts of the cities, the precariousness of the sanitation system, the deforestation and the destruction of the vector’s natural habitat, as well as the presence of the infected canine host^40^. Although the VL is of mandatory notification in Brazil, in this study which used passive secondary data, highlighted that problems of sub-notification can exist and alter the results^41^ ^,^ ^42^. 

The state of Alagoas demonstrated an accentuated geographical expansion of the HVL over the last 12 years, emphasizing the need for mass surveillance and epidemiological control, as well as health education, intrinsically connected to the knowledge of the disease, of the population.

The reassessment of the already known control measures is recommended, above all in situations in which there has been interruptions or discontinuities by the local healthcare agencies in health education strategies for the more vulnerable populations, as well as in the sanitization of the places of shelter for the animals and the areas surrounding homes; use of screens on doors and windows and of repellents at the vectors’ feeding times.

## References

[1] Sundar S, Chakravarty J (2015). An update on pharmacotherapy for leishmaniasis. Expert Opin Pharmacother.

[2] Ready PD (2014). Epidemiology of visceral leishmaniasis. Clin Epidemiol.

[3] Galati EAB, Nunes VLB, Jr FAR, Oshiro ET, Chang MR (1997). Estudo de flebotomíneos (Diptera, Psychodidae) em foco de leishmaniose visceral no estado de Mato Grosso do Sul, Brasil. Rev Saúde Pública.

[4] Santos OS, Arias JR, Ribeiro AA, Hoffmann PM, Freitas RA, Malacco MAF (1998). Incrimination of Lutzomyia (L.) cruzi as a vector of American visceral leishmaniasis. Med Vet Entomol.

[5] Gramiccia M, Gradoni L (2005). The current status of zoonotic leishmaniases and approaches to disease control. J Parasitol.

[6] Pan American Health Organization. PAHO WHO (2019). EUA: Leishmanioses Epidemiological Report of the Americas.

[7] Camargo JB, Troncarelli MZ, Ribeiro MG, Langoni H (2007). Leishmaniose visceral canina: aspectos de saúde pública e controle. Clin Vet.

[8] Reis LL, Balieiro AAS, Fonseca FR, Gonçalves MJS (2017). Changes in the epidemiology of visceral leishmaniasis in Brazil from 2001 to 2014. Rev Soc Bras Med Trop.

[9] Pan American Health Organization (2019). EUA: Leishmanioses Epidemiological Report of the Americas; 2019.

[10] Penna HA (1934). Leishmaniose visceral no Brasil. Bras Méd.

[11] Oliveira DMC, Montoni V (2003). Situação epidemiológica da leishmaniose visceral no estado de Alagoas - 2002. Rev Soc Bras Med Trop.

[12] TabNet Win32 3.0. leishmaniose visceral. Sistema de Informação de Agravos de Notificação (2019). Alagoas: Casos confirmados Notificados no Sistema de Informação de Agravos de Notificação; 2020.

[13] Secretaria de Estado da Saúde de Alagoas (SESAU) (2018). Alagoas: Nota Informativa n^º^51, GEDT, Leishmaniose Visceral em Alagoas; 2018.

[14] Ministério da Saúde (MS). Secretaria de Vigilância em Saúde. Departamento de Vigilância Epidemiológica (2014). Manual de vigilância e controle da leishmaniose visceral.

[15] Instituto Brasileiro de Geografia e Estatística (IBGE) (2010). Alagoas: Censo 2010.

[16] Barbosa DS, Belo VS, Rangel MES, Werneck GL (2014). Spatial analysis for identification of priority areas for surveillance and control in a visceral leishmaniasis endemic area in Brazil. Acta Trop.

[17] Silveira LJD, Rocha TJM, Ribeiro SA, Pedrosa CMS (2015). Historical series of patients with visceral leishmaniasis treated with meglumine antimoniate in a Hospital for Tropical Diseases, Maceió-al, Brazil. Rev Inst de Med Trop.

[18] Rocha MAN, Matos-Rocha TJ, Ribeiro CMB, Abreu SRO (2018). Epidemiological aspects of human and canine visceral leishmaniasis in State of Alagoas, Northeast, Brazil. Braz J Biol.

[19] Leite RD, Meneses RL, Magalhães TF de, Ogawa MY, Falcão H de O, Sousa A de Q (2018). Visceral Leishmaniasis hospitalizations and seasonality in Fortaleza, Ceará, Northeast Brazil between 2003 - 2012. Heal J Sci.

[20] Werneck GL (2010). Expansão geográfica da leishmaniose visceral no Brasil. Cad Saúde Pública.

[21] Oliveira-Pereira YN, Moraes JLP, Lorosa ES, Rebêlo JMM (2008). Preferência alimentar sanguínea de flebotomíneos da Amazônia do Maranhão, Brasil. Cad Saúde Pública.

[22] Rangel EF, Vilela ML (2008). Lutzomyia longipalpis (Diptera, Psychodidae, Phlebotominae) and urbanization of visceral leishmaniasis in Brazil. Cad Saúde Pública.

[23] Saraiva L, Andrade JD, Falcão AL, Carvalho DAA, Souza CM, Freitas CR (2011). Phlebotominae fauna (Diptera: Psychodidae) in an urban district of Belo Horizonte, Brazil, endemic for visceral leishmaniasis: Characterization of favored locations as determined by spatial analysis. Acta Trop.

[24] Barata RA, Peixoto JC, Tanure A, Gomes ME, Apolinário EC, Bodevan EC (2013). Epidemiology of Visceral Leishmaniasis in a Reemerging Focus of Intense Transmission in Minas Gerais State, Brazil. Bio Med Res.

[25] Lima ID, Lima ALM, Mendes-Aguiar C de O, Coutinho JFV, Wilson ME, Pearson RD (2018). Changing demographics of visceral leishmaniasis in northeast Brazil: Lessons for the future. PLoS One.

[26] Ursine RL, Dias JVL, Morais HA, Pires HHR, Ursine RL, Dias JVL (2016). Human and canine visceral leishmaniasis in an emerging focus in Araçuaí, Minas Gerais: spatial distribution and socio-environmental factors. Mem Inst Oswaldo Cruz.

[27] Vieira CP, Oliveira AM, Rodas LAC, Dibo MR, Guirado MM, Chiaravalloti F (2014). Temporal, spatial and spatiotemporal analysis of the occurrence of visceral leishmaniasis in humans in the City of Birigui, State of São Paulo, from 1999 to 2012. Rev Soc Bras Med.

[28] Xavier-Gomes LM, Costa WB, Prado PF do, Oliveira-Campos M, Leite MT de S (2009). Características clínicas e epidemiológicas da leishmaniose visceral em crianças internadas em um hospital universitário de referência no norte de Minas Gerais, Brasil. Rev Bras Epidemiol.

[29] Bevilacqua PD, Paixão HH, Modena CM, Castro MCPS (2001). Urbanização da leishmaniose visceral em Belo Horizonte. Arq Bras Med Vet.

[30] Dantas-Torres F, Brandão-Filho SP (2006). Expansão geográfica da leishmaniose visceral no Estado de Pernambuco. Rev Soc Bras Med Trop.

[31] Barbosa IR, Costa Í do CC (2013). Clinical and epidemiological aspects of visceral leishmaniasis in children up to 15 years of age in Rio Grande do Norte state, Brazil. Scientia Medica.

[32] Cardim MFM, Guirado MM, Dibo MR, Chiaravalloti F, Cardim MFM, Guirado MM (2016). Visceral leishmaniasis in the state of Sao Paulo, Brazil: spatial and space-time analysis. Rev Saúde Pública.

[33] Queiroz MJA, Alves JGB, Correia JB (2004). Leishmaniose visceral: características clínico-epidemiológicas em crianças de área endêmica. Jornal de Pediatria.

[34] Empresa Brasileira de Pesquisa Agropecuária (EMBRAPA) (2012). Alagoas: Boletim de Pesquisa e Desenvolvimento, Climatologia do Estado de Alagoas; 2012.

[35] Furtado AS, Nunes FBBF, Santos AM, Caldas AJM (2020). Análise espaço-temporal da leishmaniose visceral no estado do Maranhão, Brasil. Ciênc Saúde Colet.

[36] Pedrosa CM, Da Rocha EM (2004). Aspectos Clínico-Epidemológicos da Leishmaniose visceral em menores de 15 anos procedentes de Alagoas, Brasil. Rev Soc Bras Med Trop.

[37] Oliveira CDL, Assunção RM, Reis IA, Proietti FA (2001). Spatial distribution of human and canine visceral leishmaniasis in Belo Horizonte, Minas Gerais State, Brazil, 1994-1997. Cad Saúde Pública.

[38] Rosales JC, Yang HM (2006). Modelagem Matemática do Fator de Risco da Leishmaniose Canina na Leishmaniose Humana em Regiões Oeste do Estado de São Paulo, Brasil, e Noroeste da Província de Salta, Argentina. Researchgate.

[39] Anselin L (1995). Local indicators of spatial association - LISA. Geogr Anal.

[40] Maia CS, Pimentel DS, Santana MA, Oliveira GM, Pedrosa NA, Nascimento LA (2014). Análise espacial da Leishmaniose Visceral Americana no Município de Petrolina, Pernambuco, Brasil. Rev Bras Geo Médic Saúde.

[41] Silva TAM da, Coura-Vital W, Barbosa DS, Oiko CSF, Morais MHF, Tourinho BD (2017). Spatial and temporal trends of visceral leishmaniasis by mesoregion in a southeastern state of Brazil, 2002-2013. PLOS Negl Trop Dis.

[42] Ortiz RC, Anversa L (2015). Epidemiologia da leishmaniose visceral em Bauru, São Paulo, no período de 2004 a 2012: um estudo descritivo. Epidemiol Serv Saúde.

